# Prevalence and Factors Associated With Abnormal Cerebroplacental Ratio Among Women With Hypertensive Disorders of Pregnancy at a Tertiary Referral Hospital in Southwestern Uganda

**DOI:** 10.1155/2024/8895971

**Published:** 2024-10-17

**Authors:** Suada Suleiman Ibrahim, Yarine Fajardo Tornes, Musa Kayondo, Fidel Kasereka Tsongo, Godfrey Rwambuka Mugyenyi, Joseph Ngonzi, Henry Mark Lugobe, Julius Sebikali Mugisha, Leevan Tibaijuka

**Affiliations:** ^1^Department of Obstetrics and Gynecology, Mbarara University of Science and Technology, Mbarara, Uganda; ^2^Department of Radiology, Mbarara University of Science and Technology, Mbarara, Uganda; ^3^Department of Obstetrics and Gynecology, Mbarara Regional Referral Hospital, Mbarara, Uganda

**Keywords:** abnormal cerebroplacental ratio, hypertensive disorders of pregnancy, sub-Saharan Africa

## Abstract

**Background:** Hypertensive disorders of pregnancy (HDP) are associated with placental insufficiency and adverse perinatal outcomes—over half (58.9%) of women with HDP at Mbarara Regional Referral Hospital (MRRH) have adverse perinatal outcomes. The cerebroplacental ratio (CPR) is an important predictor and prevents approximately 30% of these adverse perinatal outcomes. We determined the prevalence and factors associated with abnormal CPR among women with HDP at MRRH.

**Methods:** We conducted a cross-sectional study from December 2022 to May 2023 at the high-risk obstetrics unit of MRRH. We consecutively enrolled all women with hypertensive disorders and gestational ages ≥ 26 weeks and performed obstetric Doppler studies to document the pulsatility index (PI) of the umbilical artery (UA) and middle cerebral artery (MCA) and then calculated the CPR as a ratio of the MCA-PI and UA-PI. The prevalence of women with an abnormal CPR ≤ 1.0 was expressed as a percentage. We used robust modified Poisson regression analysis to determine the factors associated with abnormal CPR.

**Results:** We enrolled 128 women with hypertensive disorders in pregnancy, with a mean age of 28.8 ± 6.3 years. Of these, 67 (52.3%) had abnormal CPR. The factors associated with abnormal CPR were severe pre-eclampsia (adjusted prevalence ratio (aPR): 5.0, 95% CI: 1.28, 29.14) and eclampsia (aPR: 5.27, 95% CI: 1.11, 34.27).

**Conclusion**: On average, half of the women with hypertensive disorders have abnormal CPR. Women with severe pre-eclampsia or eclampsia are more likely to have abnormal CPR. Obstetric Doppler studies with CPR may be warranted for all pregnant women with severe pre-eclampsia and eclampsia. We recommend further research to assess perinatal outcomes among those with and without abnormal CPR to profile women with HDP at increased risk of adverse perinatal outcomes.

## 1. Background

Hypertensive disorders of pregnancy (HDP) are known causes of placental insufficiency leading to adverse perinatal outcomes—over half (58.9%) of women with HDP at Mbarara Regional Referral Hospital have adverse perinatal outcomes, with 20.3% being stillbirths [1]. The cerebroplacental ratio (CPR) is an important noninvasive predictor of adverse perinatal outcomes because it considers both the umbilical artery (UA) and middle cerebral artery (MCA) Doppler velocimetry and provides valuable information about the hemodynamic status of the fetus [2–4] [5]. It has a high diagnostic accuracy in the detection of abnormal fetal well-being and can prevent approximately 30% of adverse perinatal outcomes [6]. An abnormal CPR reflects the redistribution of cardiac output to the cerebral circulation and is predictive of adverse intrapartum and neonatal outcomes. Under normal circumstances, flow in the MCA is fairly high resistance compared to that in the UA (usually low resistance), with a correspondingly lower systolic (S)/diastolic (D) ratio in the UA than in the MCA, as explained by the continuous antegrade flow and a continuous increase in D flow as pregnancy progresses [7]. However, in response to hypoxia following placental insufficiency—as occurs in HDP—the fetus diverts blood flow to the brain, increasing MCA D flow, thereby decreasing the pulsatility index and altering the ratio of MCA flow to UA flow, leading to abnormal CPR [7–9].

Despite the good predictive accuracy of CPR for adverse perinatal outcomes in HDP patients [10–12], its utility is low in our setting, as evidenced by the paucity of data on the prevalence of abnormal CPR in HDP in our setting, specifically in Uganda [13]. However, some prior studies have reported a prevalence of 29% in Kenya [14], 38% in Hamirpur, India [4], 46% in Manipur, India [15], and 46% in Mysore, India [16].

The factors reported to be associated with abnormal CPR among pregnant women with HDP include gestational age, type of HDP, birth weight, residence, and number of antenatal visits [16, 17].

Despite the important role of CPR in predicting pregnancies at increased risk of adverse perinatal outcomes and, therefore, influencing timely interventions before life-threatening fetal complications occur, CPR is not routinely performed at Mbarara Regional Referral Hospital. We, therefore, determined the prevalence and factors associated with abnormal CPR among women with HDP at Mbarara Regional Referral Hospital, Uganda.

## 2. Materials and Methods

### 2.1. Study Setting and Design

We employed a cross-sectional study design from December 2022 to May 2023 at MRRH. This study was conducted at MRRH—a government-funded public referral and teaching hospital affiliated with Mbarara University of Science and Technology located in southwestern Uganda. The maternity department performs approximately 10,000 deliveries every year and has a maternal mortality rate of 375 per 100,000 live births and a perinatal mortality rate of 33 per 1000 live births [1, 18]. The radiology department is equipped with three ultrasound scanners running color Doppler E cube 8 software and a team of radiologists and radiographers who are proficient in the ability to conduct Doppler ultrasound assessments.

MRRH has a high-risk ward dedicated to managing mothers with complicated pregnancies, making it a crucial destination for those with life-threatening medical and obstetric conditions. The hospital has a functional adult intensive care unit and a functional neonatal intensive care unit that addresses the needs of newborns with complications [19].

### 2.2. Study Population and Eligibility Criteria

We included all women with HDP and a gestational age ≥ 26 weeks admitted to the high-risk unit of Mbarara Regional Referral Hospital (MRRH). We excluded those in labor, those with intrauterine fetal deaths, and those with multiple pregnancies.

### 2.3. Sample Size Calculation and Sampling

The sample size was calculated using OpenEpi Version 3.01 [20], specifically employing sample size determination for cross-sectional studies. The calculations were based on the following assumptions: a 95% confidence level, 80% power, an exposed (gestational age < 32 weeks) to nonexposed (gestational age ≥ 32 weeks) ratio of 1:1, and a 46% occurrence of the outcome (abnormal CPR) in the exposed group. These assumptions were derived from a previous study conducted in India [16]. Considering a 10% nonresponse rate, the final sample size required for the study was determined to be 128 participants. Participants identified with HDP were consecutively sampled until the desired sample size was reached.

### 2.4. Data Collection Procedure

After obtaining written informed consent from the eligible study participants, we administered a pretested questionnaire and input data on sociodemographic, obstetric, medical and clinical characteristics into Research Electronic Data Capture (REDCap) tools—a secure, web-based software platform designed to support data capture for research studies [21]. These data included demographic information (age, residence, marital status, employment status, and referral history), obstetric information (type of HDP, gestational age, parity and number of antenatal visits, clinical symptoms, and prior history of pre-eclampsia/eclampsia), and laboratory information (platelet count and creatinine levels). After recruitment, the women underwent ultrasound plus color and pulsed Doppler imaging in the supine position with the head of the bed elevated at 45 degrees by a Philips HD6 USG machine with a 3–7 MHz transabdominal curvilinear transducer. Gestational age was calculated based on the last menstrual period or by a first trimester scan (if available). Fetal biometry and the AFI were recorded. The estimated fetal weight was calculated by ultrasound using Hadlock's formula, which included measurements of biparietal diameter (BPD), head circumference (HC), abdominal circumference (AC), and femur length (FL) [22, 23]. Doppler evaluation of the UA was performed in a free loop of the umbilical cord. The MCA was evaluated in a cross-sectional view of the fetal head angled caudally to identify the wings of the sphenoid bone, and color Doppler was used to identify the vascular circle of Willis. Spectral sampling by pulse Doppler was performed approximately 1 cm after its origin from the internal carotid artery [24]. All Doppler waveforms were recorded in the absence of fetal breathing movements. The angle of insonation was kept at < 60 degrees. The UA and MCA PIs were recorded using an automated trace of at least three consecutive waveforms. CPR was calculated as the ratio between the MCA-PI and the UA-PI. A cutoff value of 1 was used, and a CPR ≤ 1 was considered abnormal [25]. The examination bed and scan transducer (probe) were cleaned with alcohol sanitizer prior to use on the next patient to prevent transfer of infection. All the obstetric Doppler scans were conducted by the same radiologist to ensure consistency and reliability of the assessments, with every 10^th^ obstetric Doppler scan repeated by the second radiologist for quality control (all the reassessed Doppler studies for CPR were comparable).

### 2.5. Study Variables

The primary outcome was abnormal CPR, defined as CPR ≤ 1.0 [25]. The independent variables included maternal age, parity, marital status, employment status, education level, referral status, HIV serostatus, type of HDP (including gestational hypertension, pre-eclampsia, severe pre-eclampsia, eclampsia and chronic hypertension), anemia, gestational age, parity, number of antenatal visits, and prior history of pre-eclampsia. Gestational hypertension was defined as new-onset hypertension without proteinuria [26, 27]. Pre-eclampsia was defined as new-onset hypertension with proteinuria (≥ 2+ protein on dipstick) without features of severity, while severe pre-eclampsia was defined as pre-eclampsia with or without proteinuria but with severe features—including severe hypertension (S blood pressure ≥ 160 mmHg or D ≥ 110 mmHg), persistent epigastric pain, persistent headache, visual changes, elevated creatinine, and elevated liver transaminases [26, 27]. Eclampsia was defined as the presence of grand mal seizures in participants with signs and symptoms of pre-eclampsia [28]. Anemia was defined as a hemoglobin level < 11.0 g/dL [29–31].

### 2.6. Data Analysis

The data were exported to STATA 17 (StataCorp, College Station, Texas, United States) for cleaning and analysis. We described the participants' characteristics as means and standard deviations for continuous variables and as frequencies and percentages for categorical variables. The prevalence of abnormal CPR (and corresponding 95% confidence intervals) was reported as a percentage of the total study participants. To assess the factors associated with abnormal CPR among pregnant women with HDP at MRRH, we performed univariable and multivariable robust modified Poisson regression analyses and reported both crude and adjusted prevalence ratios (aPRs). In the final multivariable model, we included all exposure variables with a *p* value < 0.2 in the univariable analysis, as well as anemia and HIV serostatus based on biological plausibility. To assess collinearity in our multivariable model, we employed the variance inflation factor (VIF) method, considering a VIF > 5 to indicate collinearity. The factors associated with abnormal CPR in the multivariable model were determined based on variables with a *p* value < 0.05, indicating statistical significance.

## 3. Results

Of the 2234 women screened for inclusion in the study from December 2022 to May 2023, 167 were admitted with HDP. We excluded 39 women—19 who were in labor, 12 who experienced intrauterine fetal death, and 8 who had multiple pregnancies. We, therefore, recruited a total of 128 women with HDP.

### 3.1. Participant Characteristics

The mean age was 28.8 ± 6.3 years; the majority of participants were aged 20–34 years (75%). The highest proportion were married (90.6%), 40.6% had a primary education, and 75.0% were employed and were referred from other health facilities (64.1%). There was a significant difference (*p* < 0.05) in residence, with the majority of the participants with abnormal CPRs being from rural residences (82%) compared to those with normal CPRs (62%). The majority of participants were multigravida, had 2–4 pregnancies (54.7%), had a gestational age ≥ 34 weeks (63.8%), had ≥ 4 ANC visits, had no history of diabetes mellitus or hypertension, and were HIV seronegative. There were significant differences (*p* < 0.05) in the creatinine (mg/dL) levels and EFW, and the majority of the participants with creatinine < 1.1 mg/dL (80%) and EFW < 2500 g (59.4%) had abnormal CPR (Table 1).

### 3.2. Prevalence of Abnormal CPR Among Women With HDP at MRRH

The prevalence of abnormal CPR among pregnant women with hypertensive disorders during pregnancy at MRRH was 52.3% (95% CI: 43.6–60.9). Out of the 128 participants recruited, 67 had abnormal CPR.

### 3.3. Factors Associated With Abnormal CPR Among Women With HDP at MRRH

In the multivariable analysis (Table 2), the factors independently associated with an abnormal cerebral–placental ratio were severe pre-eclampsia and eclampsia.

Pregnant women with severe pre-eclampsia (aPR: 5.00, 95% CI: 1.28, 29.14) and eclampsia (aPR: 5.27, 95% CI: 1.11, 34.27) were more likely to have abnormal CPR than those with gestational hypertension.

## 4. Discussion

This cross-sectional study determined the prevalence and factors associated with abnormal CPR among pregnant women with hypertensive disorders at a tertiary hospital in a low-resource setting in southwestern Uganda. We found that more than half (52.3%) of the women with HDP had abnormal CPR. Additionally, severe pre-eclampsia and eclampsia were independently associated with abnormal CPR by fivefold compared to gestational hypertension. Taken together, these findings build on prior studies in our setting that have documented high rates of adverse perinatal outcomes among women with HDP [1] and highlight the need to implement routine obstetric Doppler studies with CPR assessment among women with HDP. These findings could guide the management of women with these conditions and aid in interventions to optimize perinatal outcomes.

Our study revealed an abnormal CPR in more than half of the women with HDP. This is consistent with findings from previous studies conducted in India—38% in Hamirpur [4], 46% in Manipur [15], and 46% in Mysore [16]—among a similar population of pregnant women with HDP. The high burden of abnormal CPR in our study and comparable studies could be explained by the fact that these studies involved similar populations of women with HDP. Additionally, the majority of our participants had pre-eclampsia, which is associated with abnormal placentation due to altered trophoblastic invasion—this is associated with placental insufficiency and, therefore, explains the associated abnormal CPR [32].

Our prevalence is higher than what was reported in previous studies; in Kenya (29%) [14] and India (35%) [4], these studies enrolled only women with pre-eclampsia and gestational hypertension and excluded those with eclampsia and chronic hypertension and with gestational ages > 34 weeks. This could have underestimated the burden of abnormal CPR, given the contribution of other HDP and the insult from early-onset pre-eclampsia [33], contrary to our study where all women with HDP and gestational ages ≥ 26 weeks were enrolled. The hypertension, occurring in all HDPs (even those without pre-eclampsia), is associated with increased peripheral vascular resistance due to the generalized arteriolar vasoconstriction and, hence, the consequent reduction in uteroplacental circulation; this leads to an oxygen supply deficit culminating into fetal hypoxia [34]. The persistent fetal hypoxia results in compensatory redistribution to the brain [7, 9].

In the present study, severe pre-eclampsia and eclampsia were independently associated with abnormal CPR. This is consistent with findings from other studies in Egypt [10] and Romania [35]. Pre-eclampsia is associated with abnormal placentation that eventually leads to placental insufficiency [32]. Placental insufficiency results in fetal Doppler changes, including high resistance in the UA and decreased resistance in the MCA, consequently resulting in compensatory redistribution and increased cerebral blood flow as a brain-sparing effect [7, 9]. This explains the abnormal CPR resulting from impaired placental perfusion in severe pre-eclampsia and eclampsia, which leads to a reduction in blood supply to the fetus [36].

While our study informs practice on the prevalence and factors associated with abnormal CPR at a tertiary hospital in a resource-limited setting, it was not without limitations. Our study was hospital based and at a single site, which may impact the generalizability of our findings to other settings but may be applicable to other low-resource settings with characteristics similar to ours. Our cross-sectional study design hinders the determination of causality between observed exposure factors and the development of abnormal CPR. Also, given the progressive nature of the HDP with the likelihood of worsening Doppler studies, we could have underestimated the prevalence of abnormal CPR since the Doppler studies were conducted only once for each participant.

## 5. Conclusions and Recommendations

Our study highlights a high prevalence of abnormal CPR among women with HDP. Pregnant women with severe pre-eclampsia and eclampsia are more likely to have abnormal CPR. Obstetric Doppler studies with CPR maybe considered for the assessment of pregnant women with HDP, prioritizing those with severe pre-eclampsia and eclampsia. We recommend further research to assess perinatal outcomes among those with and without abnormal CPR to profile women with HDP at increased risk of adverse perinatal outcomes, which could aid in early intervention to improve outcomes. A matched case-control study with participants matched especially in terms of gestational age may also be considered to show an association between pre-eclampsia and abnormal CPR.

## Figures and Tables

**Table 1:** Sociodemographic and clinical characteristics of women with HDP at Mbarara Regional Referral Hospital, December 2022 to May 2023.

**Characteristic**	**Total (** **N** = 128**)**	**Cerebral–placental ratio (CPR)**		**p** ** value**
		**Abnormal (** **n** = 67**)** **n (%)**	**Normal (** **n** = 61**)** **n (%)**	
Age (years) (mean ± sd)	28.77 ± 6.25	28.52 ± 5.75	29.03 ± 6.80	0.650
Maternal age category (years)				0.239
< 20	10 (7.8%)	5 (7%)	5 (8%)	
20–34	96 (75.0%)	54 (81%)	42 (69%)	
> 34	22 (17.2%)	8 (12%)	14 (23%)	
Rural residence	93 (72.7%)	55 (82%)	38 (62%)	0.012⁣^∗^
Employed	96 (75.0%)	50 (75%)	46 (75%)	0.919
Married	116 (90.6%)	61 (91%)	55 (90%)	0.860
Referred	82 (64.1%)	47 (70%)	35 (57%)	0.130
Gravidity				0.160
Primigravida (1)	32 (25.0%)	14 (21%)	18 (30%)	
Multigravida (2–4)	70 (54.7%)	42 (63%)	28 (46%)	
Grand multigravida (≥ 5)	26 (20.3%)	11 (16%)	15 (25%)	
Gestational age categories (weeks)				0.025⁣^∗^
< 34	47 (36.7%)	32 (48%)	15 (25%)	
≥ 34	81 (63.3%)	35 (52%)	46 (75%)	
ANC attendance				0.510
< 4 visits	50 (39.1%)	28 (42%)	22 (36%)	
≥ 4 visits	78 (60.9%)	39 (58%)	39 (64%)	
Type of hypertensive disorder of pregnancy				0.063
Pre-eclampsia	26 (20.3%)	13 (19%)	13 (21%)	
Severe pre-eclampsia	80 (62.5%)	46 (69%)	34 (56%)	
Eclampsia	7 (5.5%)	5 (7%)	2 (3%)	
Chronic hypertension	6 (4.7%)	2 (3%)	4 (7%)	
Gestational HTN	9 (7.0%)	1 (1%)	8 (13%)	
Prior history of pre-eclampsia	11 (8.6%)	6 (9%)	5 (8%)	0.880
Prior history of stillbirth	9 (7.0%)	5 (7%)	4 (7%)	0.840
History of diabetes	1 (0.8%)	1 (1%)	0 (0%)	0.340
Family history of hypertension	14 (10.9%)	7 (10%)	7 (11%)	0.850
Family history of pre-eclampsia	13 (10.2%)	7 (10%)	6 (10%)	0.910
HIV seropositive	6 (4.7%)	4 (6%)	2 (3%)	0.470
Symptoms at admission				
Epigastric pain	50 (39.1%)	29 (43%)	21 (34%)	0.063
Headache	72 (56.3%)	42 (63%)	30 (49%)	0.124
Blurred vision	20 (15.6%)	11 (16%)	9 (15%)	0.796
Creatinine > 1.1 mg/dL	37 (28.9%)	25 (37%)	12 (20%)	0.028⁣^∗^
Platelet count < 100,000 cells/*μ*L	34 (26.6%)	16 (24%)	18 (30%)	0.470
Anemia (hemoglobin < 11 g/dL)	27 (21.1%)	15 (22%)	12 (20%)	0.540
Estimated fetal weight < 2500 g	76 (59.4%)	48 (72%)	28 (46%)	0.730

Abbreviation: sd, standard deviation.

⁣^∗^*p* < 0.05.

**Table 2:** Factors associated with abnormal CPR among women with HDP at Mbarara Regional Referral Hospital, December 2022 to May 2023.

**Variable**	**Cerebral–placental ratio**		**Bivariate analysis**		**Multivariable analysis**	
	**Abnormal (** **n** = 67**) n (%)**	**Normal (** **n** = 61**) n (%)**	**cPR (95% CI)**	**p** ** value**	**aPR (95% CI)**	**p** ** value**
Maternal age						
< 20	5 (7%)	5 (8%)	0.89 (0.46, 1.70)	0.721	1.12 (0.60, 2.08)	0.716
20–34	54 (81%)	42 (69%)	Ref.		Ref.	
> 34	8 (12%)	14 (23%)	0.65 (0.36, 1.16)	0.142	0.70 (0.41, 1.21)	0.203
Residence						
Urban	12 (18%)	23 (38%)	Ref.		Ref.	
Rural	55 (82%)	38 (62%)	1.72 (1.06, 2.82)	0.029⁣^∗^	1.53 (0.95, 2.47)	0.082
Referral status						
No	20 (30%)	26 (43%)	Ref.		Ref.	
Yes	47 (70%)	35 (57%)	1.32 (0.90, 1.92)	0.154	1.15 (0.78, 1.70)	0.475
Gravidity						
1	14 (21%)	18 (30%)	0.73 (0.47, 1.13)	0.158	0.71 (0.46, 1.11)	0.131
2-4	42 (63%)	28 (46%)	Ref.		Ref.	
≥ 5	11 (16%)	15 (25%)	0.71 (0.47, 1.15)	0.162	0.84 (0.53, 1.33)	0.453
ANC attendance						
< 4	28 (42%)	22 (36%)	1.12 (0.80, 1.56)	0.504	1.04 (0.76, 1.41)	0.818
≥ 4	39 (58%)	39 (64%)	Ref.		Ref.	
Gestational age (weeks)						
< 34	32 (48%)	15 (25%)	1.58 (1.15, 2.17)	0.005⁣^∗^	1.26 (0.86, 1.87)	0.240
≥ 34	35 (52%)	46 (75%)	Ref.		Ref.	
Type of HDP						
Gestational hypertension	1 (1%)	8 (13%)	Ref.		Ref.	
Pre-eclampsia	13 (19%)	13 (21%)	4.49 (0.68, 29.93)	0.120	4.76 (0.84, 27.12)	0.079
Severe pre-eclampsia	46 (69%)	34 (56%)	5.17 (0.80, 33.40)	0.084	5.00 (1.28, 29.14)	0.030 ⁣^∗^
Eclampsia	5 (7%)	2 (3%)	6.43 (0.94, 43.58)	0.057	5.27 (1.11, 34.27)	0.041 ⁣^∗^
Chronic hypertension	2 (3%)	4 (7%)	2.99 (0.34, 26.42)	0.322	3.16 (0.41, 24.26)	0.269
Hemoglobin (g/dL)						
< 11	15 (22.4%)	12 (20%)	1.08 (0.73, 1.59)	0.701	1.09 (0.75, 1.58)	0.665
≥ 11	52 (77.6%)	49 (80%)	Ref.		Ref.	
HIV status						
Negative	63 (94%)	59 (97%)	Ref.		Ref.	
Positive	4 (6%)	2 (3%)	1.29 (0.71, 2.34)	0.399	0.89 (0.45, 1.77)	0.742

Abbreviations: aPR, adjusted prevalence ratio; CI, confidence interval; cPR, crude prevalence ratio; HDP, hypertensive disorder in pregnancy.

⁣^∗^* p* <0.05.

## Data Availability

The datasets used during this study are available from the corresponding author upon reasonable request.

## Nomenclature

ANC: antenatal care
CPR: cerebroplacental ratio
EFW: estimation of fetal weight
GA: gestational age
HDP: hypertensive disorders of pregnancy
HIV: human immunodeficiency virus
IUGR: intrauterine growth restriction
MCA: middle cerebral artery
MRRH: Mbarara Regional Referral Hospital
MUST: Mbarara University of Science and Technology
NICU: neonatal intensive care unit
PI: pulsatility index
REC: Research Ethics Committee
UA: umbilical artery
UNCST: Uganda National Council for Science and Technology
